# Bis[(2-pyridylmeth­yl)(triisopropyl­silyl)amido]zinc(II)–toluene–tetra­hydro­furan (4/2/1)

**DOI:** 10.1107/S160053680800888X

**Published:** 2008-04-16

**Authors:** Christian Koch, Helmar Görls, Matthias Westerhausen

**Affiliations:** a Institute of Inorganic and Analytical Chemistry, Friedrich-Schiller-Universität Jena, August-Bebel-Strasse 2, D-07743 Jena, Germany

## Abstract

The transamination reaction of (2-pyridylmeth­yl)(triiso­propyl­silyl)amine with bis­{bis­(trimethyl­silyl)amido}zinc(II) yields the colorless title solvate, [Zn(C_15_H_27_N_2_Si)_2_]·0.5C_7_H_8_·0.25C_4_H_8_O. The title compound was crystallized from toluene and tetra­hydro­furan. There are two independent mol­ecules in the asymmetric unit. In each mol­ecule, the Zn atom is tetra­hedrally coordinated by four N atoms. The two mol­ecules differ in the orientation of the isopropyl groups. The mol­ecules show large N—Zn—N angles [143.0 (2) and 145.7 (2)° between the amide groups].

## Related literature

For related literature, see: Koch *et al.* (2007 ▶), Westerhausen *et al.* (2001 ▶, 2002 ▶).
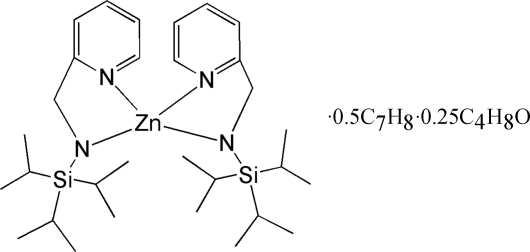


## Experimental

### 

#### Crystal data

[Zn(C_15_H_27_N_2_Si)_2_]·0.5C_7_H_8_·0.25C_4_H_8_O
*M* *_r_* = 656.44
Monoclinic, 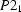

*a* = 13.2410 (3) Å
*b* = 17.6223 (4) Å
*c* = 16.7978 (5) Å
β = 91.018 (2)°
*V* = 3918.93 (17) Å^3^
*Z* = 4
Mo *K*α radiation
μ = 0.71 mm^−1^
*T* = 183 (2) K
0.04 × 0.04 × 0.03 mm

#### Data collection

Nonius KappaCCD diffractometer
Absorption correction: none
26798 measured reflections
16235 independent reflections
11581 reflections with *I* > 2σ(*I*)
*R*_int_ = 0.051

#### Refinement

*R*[*F* ^2^ > 2σ(*F* ^2^)] = 0.061
*wR*(*F* ^2^) = 0.173
*S* = 1.01
16235 reflections
754 parameters
1 restraint
H-atom parameters constrained
Δρ_max_ = 1.04 e Å^−3^
Δρ_min_ = −0.38 e Å^−3^
Absolute structure: Flack (1983 ▶), 7003 Friedel Pairs
Flack parameter: −0.026 (12)

### 

Data collection: *COLLECT* (Nonius, 1998 ▶); cell refinement: *DENZO* (Otwinowski & Minor, 1997 ▶); data reduction: *DENZO*; program(s) used to solve structure: *SHELXS97* (Sheldrick, 2008 ▶); program(s) used to refine structure: *SHELXL97* (Sheldrick, 2008 ▶); molecular graphics: *SHELXTL/PC* (Sheldrick, 2008 ▶); software used to prepare material for publication: *SHELXL97*.

## Supplementary Material

Crystal structure: contains datablocks I, global. DOI: 10.1107/S160053680800888X/fj2104sup1.cif

Structure factors: contains datablocks I. DOI: 10.1107/S160053680800888X/fj2104Isup2.hkl

Additional supplementary materials:  crystallographic information; 3D view; checkCIF report

## Figures and Tables

**Fig. 1.**
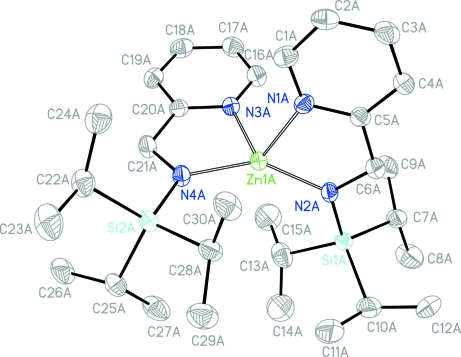
The molecular structure of 1, showing 40% probability displacement ellipsoids and the atom-numbering scheme for molecule A H atoms have been omitted for clarity.

**Fig. 2.**
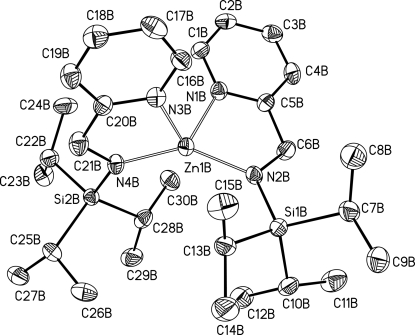
The molecular structure of 1, showing 40% probability displacement ellipsoids and the atom-numbering scheme for molecule B H atoms have been omitted for clarity.

## supplementary crystallographic information

### Comment

In the past, metallated (2-pyridylmethyl)(trialkysilyl)amines were used for oxidative C–C coupling reactions in order to prepare tetradendate bases. Zincation of (2-pyridylmethyl)(trialkylsilyl)amine (A; trialkylsilyl=*tert*BuMe_2_Si, *i*Pr_3_Si) yields dimeric methylzinc-(2-pyridylmethyl)(trialkylsilyl)amide (B). Further addition of dimethylzinc to a toluene solution of B at raised temperatures yields the C–C coupling product bis(methylzinc)-[1,2-dipyridyl-1,2-bis(trialkylsilylamido)ethane] (Westerhausen *et al.*, 2002). Thermal decomposition of B at 150°C yields [bis{(2-pyridylmethyl)(*tert*-butyldimethylsilyl)amide} zinc(II)] C (Westerhausen *et al.*, 2001, Westerhausen *et al.*, 2002). The transamination reaction of (2-pyridylmethyl)(*tert*-butylsilyl)amine with *M*[N(SiMe_3_)_2_]_2_ (*M*=Mg, Mn, Fe, Co and Zn) leads to the formation of the corresponding homoleptic [bis{(2-pyridylmethyl)(*tert*-butylsilyl)amido} metal (II)] (Koch *et al.*, 2007). The synthesis of 1 is similar (Koch *et al.*, 2007).The metal ions are in distorted tetrahedral environments. These compounds crystallize isotypically and show large N(2)–M–N(2)^'^ angles between 142° (Mg) and 156° (Mn). Due to the lower steric demand of the silyl moiety in 1 the corresponding N(2 A)–ZnA–N(4 A) angle with the value of 145.65 (19)° is smaller than 149.95 (7)° in the analogous zinc bis[(2-pyridylmethyl)(*tert*-butylsilyl)amide] C (Koch *et al.* 2007).

### Experimental

All manipulations were carried out in an atmosphere of argon using standard Schlenk techniques. Toluene, THF and pentane were dried (Na/benzophenone) and distilled prior to use. 2-Pyridylmethylamine and butyllithium were purchased form Aldrich. Triisopropylchlorosilane was purchased from Merck.^1^H-NMR and ^13^C-NMR spectra were recorded at C_6_D_6_ solution at ambient temperature on a Bruker AC 400 MHz s pectrometer and were referenced to deuterated benzene as an internal standard.

Zinc bis[(2-pyridylmethyl)(triisopropylsilyl)amide] was prepared according to a literature procedure (Koch *et al.*2007) and recrystallized from pentane, toluene and thf.

Spectroscopic data:

^1^H NMR (200 MHz, [D_6_]benzene) *δ* = 8.29 (d, ^3^*J*(H^1^,H^2^) = 6.0, 1H, Pyr1); 6.79–7.12 (m, 1H, Pyr3); 6.78 (d, ^3^*J*(H^4^,H^3^) = 7.8, 1H, Pyr4); 6.44–6.49 (m, 1H, Pyr2); 4.52 (s, 2H, CH_2_); 1.30 (s, 18H, SiCH*Me_2_*); 1.27 (s, 3H, SiC*H*Me_2_).

^13^C NMR (50 MHz, [D_6_]benzene) *δ* = 165.28 (Pyr5); 144.94 (Pyr1); 137.70 (Pyr3); 122.47 (Pyr2); 122.10 (Pyr4); 53.17 (^2^*J*, CH2); 19.59 (Si*C*HMe_2_); 14.00 (SiCH*Me_2_*).

IR (cm^-1^): 3376, 2925, 2854, 1592, 1571, 1463, 1377, 1251.

### Refinement

All hydrogen atoms were set to idealized positions and were refined with isotropic thermal parameters 1.2 times that of the attached atom (1.5 for methyl groups). The methyl groups of both molecules were allowed to rotate but not to tip. The thf molecule was modeled with a 50% occupancy.

### Crystal data

**Table:**

[Zn(C_15_H_27_N_2_Si_1_)_2_]·0.5(C_7_H_8_)·0.25(C_4_H_8_O)	*F*_000_ = 1420
*M**_r_* = 656.44	*D*_x_ = 1.113 Mg m^−^^3^
Monoclinic, *P*2_1_	Mo *K*α radiation λ = 0.71073 Å
Hall symbol: P 2yb	Cell parameters from 26798 reflections
*a* = 13.2410 (3) Å	θ = 2.3–27.5º
*b* = 17.6223 (4) Å	µ = 0.72 mm^−^^1^
*c* = 16.7978 (5) Å	*T* = 183 (2) K
β = 91.018 (2)º	Prism, colourless
*V* = 3918.93 (17) Å^3^	0.04 × 0.04 × 0.03 mm
*Z* = 4	

### Data collection

**Table:**

Nonius KappaCCD diffractometer	11581 reflections with *I* > 2σ(*I*)
Radiation source: fine-focus sealed tube	*R*_int_ = 0.051
Monochromator: graphite	θ_max_ = 27.5º
*T* = 183(2) K	θ_min_ = 2.3º
φ and ω scans	*h* = −16→17
Absorption correction: none	*k* = −22→21
26798 measured reflections	*l* = −21→21
16235 independent reflections	

### Refinement

**Table:**

Refinement on *F*^2^	Hydrogen site location: inferred from neighbouring sites
Least-squares matrix: full	H-atom parameters constrained
*R*[*F*^2^ > 2σ(*F*^2^)] = 0.061	*w* = 1/[σ^2^(*F*_o_^2^) + (0.0995*P*)^2^] where *P* = (*F*_o_^2^ + 2*F*_c_^2^)/3
*wR*(*F*^2^) = 0.173	(Δ/σ)_max_ = 0.001
*S* = 1.01	Δρ_max_ = 1.04 e Å^−^^3^
16235 reflections	Δρ_min_ = −0.38 e Å^−^^3^
754 parameters	Extinction correction: none
1 restraint	Absolute structure: Flack (1983), 7003 Friedel Pairs
Primary atom site location: structure-invariant direct methods	Flack parameter: −0.026 (12)
Secondary atom site location: difference Fourier map	

### Special details

**Table:**

Geometry. All e.s.d.'s (except the e.s.d. in the dihedral angle between two l.s. planes) are estimated using the full covariance matrix. The cell e.s.d.'s are taken into account individually in the estimation of e.s.d.'s in distances, angles and torsion angles; correlations between e.s.d.'s in cell parameters are only used when they are defined by crystal symmetry. An approximate (isotropic) treatment of cell e.s.d.'s is used for estimating e.s.d.'s involving l.s. planes.
Refinement. Refinement of *F*^2^ against ALL reflections. The weighted *R*-factor *wR* and goodness of fit *S* are based on *F*^2^, conventional *R*-factors *R* are based on *F*, with *F* set to zero for negative *F*^2^. The threshold expression of *F*^2^ > σ(*F*^2^) is used only for calculating *R*-factors(gt) *etc*. and is not relevant to the choice of reflections for refinement. *R*-factors based on *F*^2^ are statistically about twice as large as those based on *F*, and *R*- factors based on ALL data will be even larger.

### Fractional atomic coordinates and isotropic or equivalent isotropic displacement parameters (Å^2^)

**Table:**

	*x*	*y*	*z*	*U*_iso_*/*U*_eq_	Occ. (<1)
Zn1A	0.33924 (4)	1.03552 (3)	0.86223 (3)	0.02695 (15)	
Si1A	0.36403 (13)	1.01974 (8)	1.05506 (9)	0.0336 (3)	
Si2A	0.39898 (11)	1.19976 (8)	0.78840 (9)	0.0291 (3)	
N1A	0.4218 (3)	0.9452 (2)	0.8080 (3)	0.0288 (10)	
N2A	0.3895 (3)	0.9918 (2)	0.9601 (3)	0.0303 (10)	
N3A	0.1902 (3)	1.0067 (2)	0.8238 (3)	0.0283 (9)	
N4A	0.3154 (3)	1.1277 (2)	0.8040 (3)	0.0295 (10)	
C1A	0.4342 (4)	0.9266 (3)	0.7307 (3)	0.0361 (13)	
H1AA	0.4037	0.9576	0.6908	0.043*	
C2A	0.4888 (4)	0.8654 (3)	0.7081 (4)	0.0386 (14)	
H2AA	0.4946	0.8532	0.6533	0.046*	
C3A	0.5357 (4)	0.8209 (3)	0.7653 (4)	0.0391 (14)	
H3AA	0.5751	0.7784	0.7505	0.047*	
C4A	0.5242 (4)	0.8395 (3)	0.8452 (4)	0.0375 (13)	
H4AA	0.5549	0.8094	0.8859	0.045*	
C5A	0.4672 (4)	0.9027 (3)	0.8644 (3)	0.0294 (11)	
C6A	0.4554 (4)	0.9278 (3)	0.9496 (3)	0.0364 (13)	
H6AA	0.4291	0.8846	0.9807	0.044*	
H6AB	0.5229	0.9408	0.9719	0.044*	
C7A	0.3222 (5)	0.9345 (3)	1.1157 (3)	0.0392 (14)	
H7AA	0.3820	0.8999	1.1185	0.047*	
C8A	0.2971 (8)	0.9531 (4)	1.2026 (4)	0.074 (3)	
H8AA	0.2888	0.9059	1.2325	0.111*	
H8AB	0.2342	0.9825	1.2040	0.111*	
H8AC	0.3521	0.9829	1.2267	0.111*	
C9A	0.2378 (5)	0.8886 (4)	1.0755 (4)	0.0481 (16)	
H9AA	0.2219	0.8445	1.1085	0.072*	
H9AB	0.2598	0.8714	1.0231	0.072*	
H9AC	0.1775	0.9204	1.0691	0.072*	
C10A	0.4798 (6)	1.0600 (4)	1.1099 (4)	0.0517 (17)	
H10A	0.4551	1.0833	1.1602	0.062*	
C11A	0.5289 (7)	1.1234 (5)	1.0628 (6)	0.077 (3)	
H11A	0.5757	1.1516	1.0976	0.115*	
H11B	0.4766	1.1579	1.0422	0.115*	
H11C	0.5660	1.1016	1.0183	0.115*	
C12A	0.5593 (7)	1.0014 (5)	1.1341 (5)	0.073 (2)	
H12A	0.6179	1.0271	1.1582	0.109*	
H12B	0.5804	0.9731	1.0870	0.109*	
H12C	0.5307	0.9661	1.1729	0.109*	
C13A	0.2666 (6)	1.0980 (4)	1.0475 (4)	0.0514 (17)	
H13A	0.2860	1.1296	1.0007	0.062*	
C14A	0.2652 (7)	1.1533 (5)	1.1203 (5)	0.072 (2)*	
H14A	0.2125	1.1916	1.1117	0.107*	
H14B	0.3310	1.1783	1.1259	0.107*	
H14C	0.2512	1.1245	1.1688	0.107*	
C15A	0.1574 (6)	1.0723 (5)	1.0309 (5)	0.069 (2)*	
H15A	0.1152	1.1167	1.0190	0.104*	
H15B	0.1315	1.0462	1.0778	0.104*	
H15C	0.1557	1.0377	0.9852	0.104*	
C16A	0.1368 (4)	0.9448 (3)	0.8358 (4)	0.0383 (13)	
H16A	0.1669	0.9045	0.8653	0.046*	
C17A	0.0373 (5)	0.9358 (4)	0.8068 (4)	0.0420 (14)	
H17A	−0.0006	0.8914	0.8178	0.050*	
C18A	−0.0032 (4)	0.9943 (4)	0.7615 (4)	0.0426 (15)	
H18A	−0.0693	0.9896	0.7390	0.051*	
C19A	0.0515 (4)	1.0587 (3)	0.7490 (3)	0.0373 (13)	
H19A	0.0233	1.0995	0.7192	0.045*	
C20A	0.1511 (4)	1.0638 (3)	0.7812 (3)	0.0297 (11)	
C21A	0.2143 (4)	1.1347 (3)	0.7703 (4)	0.0348 (13)	
H21A	0.2190	1.1459	0.7127	0.042*	
H21B	0.1799	1.1781	0.7957	0.042*	
C22A	0.4036 (5)	1.2254 (4)	0.6770 (3)	0.0423 (14)	
H22A	0.3369	1.2495	0.6642	0.051*	
C23A	0.4814 (6)	1.2841 (4)	0.6574 (5)	0.062 (2)	
H23A	0.4769	1.2962	0.6005	0.094*	
H23B	0.5489	1.2644	0.6704	0.094*	
H23C	0.4691	1.3302	0.6885	0.094*	
C24A	0.4098 (5)	1.1557 (4)	0.6219 (4)	0.0495 (16)	
H24A	0.3926	1.1708	0.5672	0.074*	
H24B	0.3622	1.1169	0.6395	0.074*	
H24C	0.4785	1.1352	0.6239	0.074*	
C25A	0.3677 (4)	1.2927 (3)	0.8403 (4)	0.0367 (13)	
H25A	0.4292	1.3256	0.8395	0.044*	
C26A	0.2796 (5)	1.3372 (4)	0.8017 (4)	0.0509 (17)	
H26A	0.2645	1.3818	0.8343	0.076*	
H26B	0.2198	1.3046	0.7980	0.076*	
H26C	0.2984	1.3536	0.7482	0.076*	
C27A	0.3398 (6)	1.2793 (4)	0.9278 (4)	0.0547 (18)	
H27A	0.3273	1.3282	0.9536	0.082*	
H27B	0.3956	1.2533	0.9555	0.082*	
H27C	0.2788	1.2480	0.9299	0.082*	
C28A	0.5249 (4)	1.1648 (3)	0.8311 (4)	0.0374 (13)	
H28A	0.5080	1.1331	0.8785	0.045*	
C29A	0.5978 (5)	1.2263 (4)	0.8620 (5)	0.0540 (17)	
H29A	0.6590	1.2024	0.8841	0.081*	
H29B	0.5651	1.2560	0.9036	0.081*	
H29C	0.6160	1.2599	0.8181	0.081*	
C30A	0.5805 (4)	1.1120 (4)	0.7742 (4)	0.0474 (16)	
H30A	0.6445	1.0959	0.7990	0.071*	
H30B	0.5940	1.1391	0.7246	0.071*	
H30C	0.5386	1.0674	0.7627	0.071*	
Zn1B	0.16087 (4)	0.72556 (3)	0.63234 (3)	0.02721 (15)	
Si1B	−0.07885 (10)	0.70358 (8)	0.59155 (9)	0.0284 (3)	
Si2B	0.31298 (11)	0.59836 (8)	0.55680 (9)	0.0267 (3)	
N1B	0.1792 (3)	0.7365 (3)	0.7578 (3)	0.0299 (10)	
N2B	0.0210 (3)	0.7084 (3)	0.6574 (2)	0.0290 (9)	
N3B	0.2153 (3)	0.8345 (2)	0.5982 (3)	0.0288 (10)	
N4B	0.2689 (3)	0.6892 (2)	0.5682 (3)	0.0297 (10)	
C1B	0.2603 (4)	0.7557 (3)	0.8026 (4)	0.0355 (13)	
H1BA	0.3217	0.7666	0.7765	0.043*	
C2B	0.2587 (5)	0.7604 (3)	0.8840 (4)	0.0371 (13)	
H2BA	0.3174	0.7745	0.9137	0.045*	
C3B	0.1681 (5)	0.7437 (3)	0.9223 (3)	0.0392 (14)	
H3BA	0.1643	0.7454	0.9787	0.047*	
C4B	0.0845 (4)	0.7249 (3)	0.8760 (3)	0.0376 (13)	
H4BA	0.0219	0.7144	0.9007	0.045*	
C5B	0.0914 (4)	0.7212 (3)	0.7940 (3)	0.0291 (11)	
C6B	0.0033 (4)	0.6978 (4)	0.7420 (3)	0.0363 (13)	
H6BA	−0.0118	0.6436	0.7519	0.044*	
H6BB	−0.0567	0.7277	0.7570	0.044*	
C7B	−0.1881 (4)	0.7642 (4)	0.6286 (4)	0.0413 (14)	
H7BA	−0.2121	0.7393	0.6783	0.050*	
C8B	−0.1581 (5)	0.8458 (4)	0.6520 (4)	0.0527 (17)*	
H8BA	−0.2125	0.8690	0.6824	0.079*	
H8BB	−0.1465	0.8756	0.6038	0.079*	
H8BC	−0.0960	0.8446	0.6847	0.079*	
C9B	−0.2801 (5)	0.7663 (4)	0.5705 (4)	0.0527 (18)	
H9BA	−0.3357	0.7939	0.5951	0.079*	
H9BB	−0.3018	0.7143	0.5583	0.079*	
H9BC	−0.2612	0.7919	0.5212	0.079*	
C10B	−0.1340 (4)	0.6054 (3)	0.5768 (4)	0.0374 (13)	
H10B	−0.1807	0.6078	0.5295	0.045*	
C11B	−0.1974 (5)	0.5763 (4)	0.6491 (4)	0.0550 (18)	
H11D	−0.2194	0.5240	0.6390	0.083*	
H11E	−0.2567	0.6089	0.6555	0.083*	
H11F	−0.1556	0.5780	0.6978	0.083*	
C12B	−0.0527 (5)	0.5458 (4)	0.5587 (5)	0.0550 (18)	
H12D	−0.0838	0.4954	0.5558	0.082*	
H12E	−0.0008	0.5463	0.6010	0.082*	
H12F	−0.0216	0.5577	0.5076	0.082*	
C13B	−0.0279 (4)	0.7355 (3)	0.4918 (3)	0.0360 (13)	
H13B	0.0408	0.7123	0.4882	0.043*	
C14B	−0.0876 (5)	0.7070 (5)	0.4170 (4)	0.059 (2)	
H14D	−0.0551	0.7257	0.3688	0.088*	
H14E	−0.1571	0.7259	0.4185	0.088*	
H14F	−0.0882	0.6514	0.4165	0.088*	
C15B	−0.0116 (6)	0.8202 (4)	0.4862 (4)	0.0530 (17)	
H15D	0.0324	0.8313	0.4413	0.080*	
H15E	0.0202	0.8386	0.5356	0.080*	
H15F	−0.0768	0.8456	0.4779	0.080*	
C16B	0.1863 (4)	0.9045 (3)	0.6184 (3)	0.0355 (13)	
H16B	0.1341	0.9096	0.6560	0.043*	
C17B	0.2275 (5)	0.9694 (3)	0.5879 (4)	0.0409 (14)	
H17B	0.2054	1.0181	0.6046	0.049*	
C18B	0.3011 (5)	0.9615 (3)	0.5330 (4)	0.0389 (14)	
H18B	0.3307	1.0052	0.5098	0.047*	
C19B	0.3321 (4)	0.8916 (3)	0.5115 (4)	0.0382 (14)	
H19B	0.3838	0.8860	0.4734	0.046*	
C20B	0.2881 (4)	0.8274 (3)	0.5451 (3)	0.0301 (11)	
C21B	0.3212 (5)	0.7483 (3)	0.5243 (4)	0.0395 (14)	
H21C	0.3094	0.7401	0.4666	0.047*	
H21D	0.3947	0.7437	0.5351	0.047*	
C22B	0.4494 (4)	0.5906 (3)	0.5924 (4)	0.0370 (13)	
H22B	0.4925	0.6080	0.5476	0.044*	
C23B	0.4844 (4)	0.5098 (4)	0.6148 (4)	0.0450 (15)	
H23D	0.5572	0.5101	0.6264	0.067*	
H23E	0.4699	0.4752	0.5704	0.067*	
H23F	0.4483	0.4927	0.6620	0.067*	
C24B	0.4734 (5)	0.6428 (4)	0.6644 (4)	0.0487 (16)	
H24D	0.5461	0.6415	0.6764	0.073*	
H24E	0.4363	0.6250	0.7109	0.073*	
H24F	0.4529	0.6949	0.6517	0.073*	
C25B	0.3109 (4)	0.5733 (3)	0.4459 (3)	0.0346 (12)	
H25B	0.3441	0.6174	0.4196	0.041*	
C26B	0.2035 (5)	0.5727 (4)	0.4112 (4)	0.0505 (16)	
H26D	0.2059	0.5777	0.3531	0.076*	
H26E	0.1653	0.6152	0.4332	0.076*	
H26F	0.1705	0.5248	0.4249	0.076*	
C27B	0.3714 (5)	0.5042 (4)	0.4192 (4)	0.0442 (15)	
H27D	0.3622	0.4972	0.3616	0.066*	
H27E	0.3476	0.4589	0.4470	0.066*	
H27F	0.4432	0.5122	0.4318	0.066*	
C28B	0.2242 (4)	0.5346 (3)	0.6128 (3)	0.0360 (12)	
H28B	0.1547	0.5518	0.5969	0.043*	
C29B	0.2278 (6)	0.4504 (4)	0.5911 (4)	0.0517 (18)	
H29D	0.1793	0.4224	0.6232	0.078*	
H29E	0.2960	0.4308	0.6015	0.078*	
H29F	0.2105	0.4442	0.5345	0.078*	
C30B	0.2298 (5)	0.5435 (4)	0.7044 (3)	0.0426 (14)	
H30D	0.1673	0.5246	0.7276	0.064*	
H30E	0.2386	0.5972	0.7180	0.064*	
H30F	0.2873	0.5144	0.7257	0.064*	
C1T	0.8334 (9)	0.9809 (6)	0.9947 (7)	0.095 (3)	
C2T	0.7634 (8)	0.9935 (6)	0.9348 (8)	0.107 (4)	
H2TC	0.7270	1.0399	0.9334	0.128*	
C3T	0.7458 (7)	0.9415 (6)	0.8788 (8)	0.098 (3)	
H3TC	0.7008	0.9534	0.8359	0.118*	
C4T	0.7899 (8)	0.8722 (6)	0.8807 (7)	0.090 (3)	
H4TC	0.7739	0.8353	0.8413	0.108*	
C5T	0.8572 (8)	0.8567 (6)	0.9402 (7)	0.095 (3)	
H5TA	0.8879	0.8080	0.9439	0.114*	
C6T	0.8809 (7)	0.9125 (5)	0.9957 (7)	0.083 (3)	
H6TA	0.9314	0.9027	1.0352	0.099*	
C7T	0.8582 (12)	1.0359 (10)	1.0535 (10)	0.160 (6)*	
H7TA	0.9090	1.0150	1.0905	0.240*	
H7TB	0.7974	1.0494	1.0828	0.240*	
H7TC	0.8853	1.0813	1.0278	0.240*	
O1TH	0.8602 (9)	1.2011 (7)	0.7855 (7)	0.079 (3)*	0.50
C1TH	0.9455 (13)	1.2052 (10)	0.8595 (11)	0.075 (5)*	0.50
H1TA	0.9120	1.2047	0.9117	0.090*	0.50
H1TB	0.9925	1.1616	0.8570	0.090*	0.50
C2TH	0.9996 (9)	1.2771 (7)	0.8477 (8)	0.042 (3)*	0.50
H2TA	1.0724	1.2669	0.8404	0.050*	0.50
H2TB	0.9924	1.3097	0.8952	0.050*	0.50
C3TH	0.9563 (9)	1.3178 (7)	0.7743 (7)	0.040 (3)*	0.50
H3TA	0.9321	1.3686	0.7903	0.048*	0.50
H3TB	1.0108	1.3250	0.7355	0.048*	0.50
C4TH	0.8709 (11)	1.2755 (8)	0.7348 (9)	0.057 (4)*	0.50
H4TA	0.8868	1.2634	0.6789	0.068*	0.50
H4TB	0.8079	1.3057	0.7357	0.068*	0.50

### Atomic displacement parameters (Å^2^)

**Table:**

	*U*^11^	*U*^22^	*U*^33^	*U*^12^	*U*^13^	*U*^23^
Zn1A	0.0295 (3)	0.0243 (3)	0.0270 (3)	0.0012 (3)	−0.0014 (2)	0.0016 (3)
Si1A	0.0493 (9)	0.0249 (8)	0.0266 (8)	0.0012 (7)	−0.0030 (7)	−0.0014 (6)
Si2A	0.0300 (7)	0.0240 (7)	0.0333 (8)	−0.0005 (6)	0.0027 (6)	0.0021 (6)
N1A	0.027 (2)	0.031 (2)	0.028 (2)	0.0009 (19)	−0.0030 (19)	−0.0012 (19)
N2A	0.036 (2)	0.027 (2)	0.028 (2)	0.0079 (19)	−0.0021 (19)	0.0007 (18)
N3A	0.030 (2)	0.026 (2)	0.028 (2)	0.0000 (19)	−0.0005 (18)	0.0020 (18)
N4A	0.027 (2)	0.028 (2)	0.034 (3)	−0.0008 (18)	−0.0018 (19)	0.0066 (19)
C1A	0.039 (3)	0.041 (3)	0.028 (3)	−0.002 (3)	0.004 (2)	−0.002 (3)
C2A	0.039 (3)	0.041 (3)	0.036 (3)	−0.002 (3)	0.005 (3)	−0.012 (3)
C3A	0.037 (3)	0.037 (3)	0.043 (4)	0.008 (3)	0.005 (3)	−0.010 (3)
C4A	0.042 (3)	0.030 (3)	0.040 (3)	0.009 (3)	−0.005 (3)	−0.005 (2)
C5A	0.025 (2)	0.029 (3)	0.034 (3)	−0.001 (2)	−0.002 (2)	−0.003 (2)
C6A	0.042 (3)	0.031 (3)	0.036 (3)	0.007 (3)	−0.004 (3)	−0.001 (2)
C7A	0.056 (4)	0.035 (3)	0.027 (3)	0.002 (3)	0.001 (3)	−0.003 (2)
C8A	0.141 (8)	0.053 (5)	0.029 (4)	−0.006 (5)	0.012 (4)	0.000 (3)
C9A	0.060 (4)	0.040 (4)	0.044 (4)	−0.008 (3)	0.013 (3)	0.007 (3)
C10A	0.076 (5)	0.041 (4)	0.038 (4)	−0.009 (3)	−0.009 (3)	−0.002 (3)
C11A	0.086 (6)	0.056 (5)	0.087 (6)	−0.026 (4)	−0.037 (5)	0.009 (4)
C12A	0.083 (5)	0.058 (4)	0.076 (6)	−0.005 (4)	−0.051 (5)	−0.003 (4)
C13A	0.074 (5)	0.038 (3)	0.042 (4)	0.009 (3)	0.015 (3)	0.005 (3)
C16A	0.038 (3)	0.033 (3)	0.044 (3)	0.002 (3)	0.000 (3)	0.003 (3)
C17A	0.043 (3)	0.037 (3)	0.046 (4)	−0.007 (3)	0.003 (3)	−0.001 (3)
C18A	0.031 (3)	0.054 (4)	0.043 (4)	0.001 (3)	−0.005 (3)	−0.008 (3)
C19A	0.035 (3)	0.042 (3)	0.035 (3)	0.003 (3)	−0.003 (2)	0.001 (3)
C20A	0.029 (3)	0.031 (3)	0.029 (3)	0.001 (2)	−0.001 (2)	−0.004 (2)
C21A	0.031 (3)	0.030 (3)	0.043 (3)	−0.001 (2)	−0.003 (2)	0.009 (3)
C22A	0.047 (3)	0.042 (3)	0.038 (3)	0.003 (3)	0.011 (3)	0.006 (3)
C23A	0.070 (5)	0.061 (5)	0.057 (5)	−0.008 (4)	0.023 (4)	0.020 (4)
C24A	0.053 (4)	0.057 (4)	0.038 (4)	0.009 (3)	0.009 (3)	0.003 (3)
C25A	0.038 (3)	0.028 (3)	0.043 (3)	0.004 (2)	0.007 (3)	−0.002 (3)
C26A	0.054 (4)	0.031 (3)	0.067 (5)	0.016 (3)	−0.001 (3)	−0.004 (3)
C27A	0.076 (5)	0.038 (3)	0.051 (4)	−0.001 (3)	0.019 (4)	−0.010 (3)
C28A	0.033 (3)	0.029 (3)	0.050 (4)	−0.002 (2)	−0.005 (3)	−0.004 (3)
C29A	0.042 (3)	0.047 (4)	0.073 (5)	−0.003 (3)	−0.007 (3)	−0.009 (4)
C30A	0.033 (3)	0.039 (3)	0.069 (5)	0.009 (3)	−0.002 (3)	−0.005 (3)
Zn1B	0.0253 (3)	0.0298 (3)	0.0266 (3)	0.0001 (3)	0.0029 (2)	0.0016 (3)
Si1B	0.0259 (7)	0.0312 (8)	0.0282 (7)	−0.0013 (6)	0.0005 (6)	−0.0007 (6)
Si2B	0.0284 (7)	0.0260 (7)	0.0259 (7)	−0.0008 (6)	0.0032 (6)	0.0002 (6)
N1B	0.028 (2)	0.034 (2)	0.028 (2)	0.0022 (19)	0.0008 (18)	0.0016 (19)
N2B	0.028 (2)	0.039 (3)	0.020 (2)	0.0020 (19)	0.0014 (17)	−0.0004 (19)
N3B	0.030 (2)	0.027 (2)	0.029 (2)	0.0024 (19)	−0.0003 (19)	0.0031 (19)
N4B	0.031 (2)	0.030 (2)	0.029 (2)	−0.0008 (19)	0.0068 (19)	0.0062 (19)
C1B	0.029 (3)	0.034 (3)	0.043 (3)	−0.004 (2)	−0.001 (2)	−0.004 (3)
C2B	0.040 (3)	0.034 (3)	0.037 (3)	−0.003 (3)	−0.010 (3)	−0.005 (3)
C3B	0.051 (3)	0.039 (3)	0.028 (3)	−0.006 (3)	0.001 (3)	−0.001 (2)
C4B	0.040 (3)	0.041 (3)	0.032 (3)	0.002 (3)	0.013 (2)	0.007 (3)
C5B	0.029 (2)	0.029 (3)	0.030 (3)	−0.002 (2)	0.001 (2)	0.005 (2)
C6B	0.031 (3)	0.054 (4)	0.024 (3)	−0.007 (3)	−0.001 (2)	0.004 (3)
C7B	0.035 (3)	0.049 (4)	0.040 (3)	0.010 (3)	−0.005 (3)	−0.012 (3)
C9B	0.038 (3)	0.060 (4)	0.060 (4)	0.017 (3)	−0.006 (3)	−0.012 (4)
C10B	0.030 (3)	0.045 (3)	0.037 (3)	−0.007 (3)	−0.005 (2)	0.000 (3)
C11B	0.057 (4)	0.060 (4)	0.047 (4)	−0.024 (4)	−0.001 (3)	0.011 (3)
C12B	0.045 (4)	0.041 (4)	0.078 (5)	0.003 (3)	−0.004 (3)	−0.014 (3)
C13B	0.039 (3)	0.040 (3)	0.029 (3)	−0.006 (3)	0.001 (2)	−0.004 (3)
C14B	0.059 (4)	0.086 (6)	0.031 (3)	−0.019 (4)	−0.001 (3)	0.001 (4)
C15B	0.062 (4)	0.049 (4)	0.047 (4)	−0.004 (3)	−0.010 (3)	0.011 (3)
C16B	0.037 (3)	0.034 (3)	0.035 (3)	0.004 (3)	0.003 (2)	−0.006 (2)
C17B	0.054 (4)	0.032 (3)	0.037 (3)	0.007 (3)	−0.003 (3)	−0.004 (3)
C18B	0.046 (3)	0.030 (3)	0.041 (3)	−0.004 (3)	0.001 (3)	0.007 (3)
C19B	0.041 (3)	0.035 (3)	0.038 (3)	0.002 (3)	0.009 (3)	0.007 (3)
C20B	0.026 (3)	0.031 (3)	0.033 (3)	0.002 (2)	−0.001 (2)	0.002 (2)
C21B	0.041 (3)	0.036 (3)	0.042 (3)	0.001 (3)	0.010 (3)	0.007 (3)
C22B	0.034 (3)	0.035 (3)	0.042 (3)	0.003 (2)	−0.001 (3)	−0.005 (3)
C23B	0.036 (3)	0.052 (4)	0.047 (4)	0.019 (3)	−0.004 (3)	0.005 (3)
C24B	0.041 (3)	0.045 (4)	0.059 (4)	0.003 (3)	−0.016 (3)	−0.017 (3)
C25B	0.040 (3)	0.033 (3)	0.030 (3)	0.003 (2)	0.003 (2)	−0.006 (2)
C26B	0.060 (4)	0.058 (4)	0.033 (3)	−0.003 (3)	−0.011 (3)	−0.008 (3)
C27B	0.056 (4)	0.041 (3)	0.036 (3)	−0.002 (3)	0.002 (3)	−0.007 (3)
C28B	0.032 (3)	0.042 (3)	0.035 (3)	−0.009 (3)	−0.001 (2)	0.009 (3)
C29B	0.062 (4)	0.049 (4)	0.044 (4)	−0.028 (3)	0.006 (3)	0.001 (3)
C30B	0.055 (4)	0.039 (3)	0.034 (3)	0.001 (3)	0.007 (3)	0.003 (3)
C1T	0.104 (8)	0.078 (7)	0.104 (8)	−0.032 (6)	0.027 (7)	−0.021 (6)
C2T	0.081 (7)	0.068 (6)	0.169 (12)	−0.011 (5)	−0.042 (8)	−0.005 (7)
C3T	0.066 (6)	0.083 (7)	0.145 (11)	−0.016 (5)	−0.022 (6)	0.007 (7)
C4T	0.084 (7)	0.093 (8)	0.092 (8)	−0.003 (6)	0.009 (6)	0.003 (6)
C5T	0.093 (7)	0.078 (7)	0.114 (9)	0.012 (6)	0.032 (7)	−0.005 (6)
C6T	0.081 (6)	0.066 (6)	0.102 (8)	0.011 (5)	0.016 (5)	0.016 (5)

### Geometric parameters (Å, °)

**Table:**

Zn1A—N4A	1.920 (4)	N2B—C6B	1.456 (6)
Zn1A—N2A	1.924 (4)	N3B—C20B	1.330 (7)
Zn1A—N3A	2.127 (4)	N3B—C16B	1.338 (7)
Zn1A—N1A	2.143 (4)	N4B—C21B	1.458 (7)
Si1A—N2A	1.709 (5)	C1B—C2B	1.371 (8)
Si1A—C13A	1.891 (7)	C1B—H1BA	0.9500
Si1A—C7A	1.902 (6)	C2B—C3B	1.402 (8)
Si1A—C10A	1.911 (7)	C2B—H2BA	0.9500
Si2A—N4A	1.708 (4)	C3B—C4B	1.382 (8)
Si2A—C25A	1.904 (6)	C3B—H3BA	0.9500
Si2A—C28A	1.906 (6)	C4B—C5B	1.385 (7)
Si2A—C22A	1.926 (6)	C4B—H4BA	0.9500
N1A—C5A	1.341 (7)	C5B—C6B	1.503 (7)
N1A—C1A	1.352 (7)	C6B—H6BA	0.9900
N2A—C6A	1.438 (7)	C6B—H6BB	0.9900
N3A—C16A	1.317 (7)	C7B—C8B	1.541 (9)
N3A—C20A	1.335 (7)	C7B—C9B	1.547 (8)
N4A—C21A	1.449 (7)	C7B—H7BA	1.0000
C1A—C2A	1.357 (8)	C8B—H8BA	0.9800
C1A—H1AA	0.9500	C8B—H8BB	0.9800
C2A—C3A	1.379 (8)	C8B—H8BC	0.9800
C2A—H2AA	0.9500	C9B—H9BA	0.9800
C3A—C4A	1.392 (8)	C9B—H9BB	0.9800
C3A—H3AA	0.9500	C9B—H9BC	0.9800
C4A—C5A	1.387 (8)	C10B—C12B	1.539 (9)
C4A—H4AA	0.9500	C10B—C11B	1.574 (9)
C5A—C6A	1.508 (8)	C10B—H10B	1.0000
C6A—H6AA	0.9900	C11B—H11D	0.9800
C6A—H6AB	0.9900	C11B—H11E	0.9800
C7A—C9A	1.528 (9)	C11B—H11F	0.9800
C7A—C8A	1.539 (9)	C12B—H12D	0.9800
C7A—H7AA	1.0000	C12B—H12E	0.9800
C8A—H8AA	0.9800	C12B—H12F	0.9800
C8A—H8AB	0.9800	C13B—C15B	1.511 (9)
C8A—H8AC	0.9800	C13B—C14B	1.556 (8)
C9A—H9AA	0.9800	C13B—H13B	1.0000
C9A—H9AB	0.9800	C14B—H14D	0.9800
C9A—H9AC	0.9800	C14B—H14E	0.9800
C10A—C11A	1.522 (10)	C14B—H14F	0.9800
C10A—C12A	1.525 (10)	C15B—H15D	0.9800
C10A—H10A	1.0000	C15B—H15E	0.9800
C11A—H11A	0.9800	C15B—H15F	0.9800
C11A—H11B	0.9800	C16B—C17B	1.368 (9)
C11A—H11C	0.9800	C16B—H16B	0.9500
C12A—H12A	0.9800	C17B—C18B	1.362 (9)
C12A—H12B	0.9800	C17B—H17B	0.9500
C12A—H12C	0.9800	C18B—C19B	1.351 (8)
C13A—C15A	1.537 (11)	C18B—H18B	0.9500
C13A—C14A	1.563 (11)	C19B—C20B	1.396 (8)
C13A—H13A	1.0000	C19B—H19B	0.9500
C14A—H14A	0.9800	C20B—C21B	1.504 (8)
C14A—H14B	0.9800	C21B—H21C	0.9900
C14A—H14C	0.9800	C21B—H21D	0.9900
C15A—H15A	0.9800	C22B—C23B	1.543 (8)
C15A—H15B	0.9800	C22B—C24B	1.549 (8)
C15A—H15C	0.9800	C22B—H22B	1.0000
C16A—C17A	1.406 (9)	C23B—H23D	0.9800
C16A—H16A	0.9500	C23B—H23E	0.9800
C17A—C18A	1.384 (9)	C23B—H23F	0.9800
C17A—H17A	0.9500	C24B—H24D	0.9800
C18A—C19A	1.364 (9)	C24B—H24E	0.9800
C18A—H18A	0.9500	C24B—H24F	0.9800
C19A—C20A	1.419 (8)	C25B—C26B	1.526 (9)
C19A—H19A	0.9500	C25B—C27B	1.530 (8)
C20A—C21A	1.516 (8)	C25B—H25B	1.0000
C21A—H21A	0.9900	C26B—H26D	0.9800
C21A—H21B	0.9900	C26B—H26E	0.9800
C22A—C23A	1.502 (9)	C26B—H26F	0.9800
C22A—C24A	1.540 (9)	C27B—H27D	0.9800
C22A—H22A	1.0000	C27B—H27E	0.9800
C23A—H23A	0.9800	C27B—H27F	0.9800
C23A—H23B	0.9800	C28B—C29B	1.529 (9)
C23A—H23C	0.9800	C28B—C30B	1.548 (8)
C24A—H24A	0.9800	C28B—H28B	1.0000
C24A—H24B	0.9800	C29B—H29D	0.9800
C24A—H24C	0.9800	C29B—H29E	0.9800
C25A—C26A	1.539 (8)	C29B—H29F	0.9800
C25A—C27A	1.540 (9)	C30B—H30D	0.9800
C25A—H25A	1.0000	C30B—H30E	0.9800
C26A—H26A	0.9800	C30B—H30F	0.9800
C26A—H26B	0.9800	C1T—C6T	1.361 (14)
C26A—H26C	0.9800	C1T—C2T	1.373 (15)
C27A—H27A	0.9800	C1T—C7T	1.418 (17)
C27A—H27B	0.9800	C2T—C3T	1.332 (15)
C27A—H27C	0.9800	C2T—H2TC	0.9500
C28A—C30A	1.532 (8)	C3T—C4T	1.352 (14)
C28A—C29A	1.537 (8)	C3T—H3TC	0.9500
C28A—H28A	1.0000	C4T—C5T	1.356 (14)
C29A—H29A	0.9800	C4T—H4TC	0.9500
C29A—H29B	0.9800	C5T—C6T	1.386 (14)
C29A—H29C	0.9800	C5T—H5TA	0.9500
C30A—H30A	0.9800	C6T—H6TA	0.9500
C30A—H30B	0.9800	C7T—H7TA	0.9800
C30A—H30C	0.9800	C7T—H7TB	0.9800
Zn1B—N4B	1.916 (4)	C7T—H7TC	0.9800
Zn1B—N2B	1.930 (4)	O1TH—C4TH	1.570 (18)
Zn1B—N1B	2.126 (4)	O1TH—C1TH	1.67 (2)
Zn1B—N3B	2.133 (4)	C1TH—C2TH	1.47 (2)
Si1B—N2B	1.711 (4)	C1TH—H1TA	0.9900
Si1B—C10B	1.893 (6)	C1TH—H1TB	0.9900
Si1B—C13B	1.903 (6)	C2TH—C3TH	1.528 (17)
Si1B—C7B	1.911 (6)	C2TH—H2TA	0.9900
Si2B—N4B	1.716 (5)	C2TH—H2TB	0.9900
Si2B—C28B	1.889 (5)	C3TH—C4TH	1.499 (18)
Si2B—C22B	1.898 (6)	C3TH—H3TA	0.9900
Si2B—C25B	1.914 (6)	C3TH—H3TB	0.9900
N1B—C1B	1.343 (7)	C4TH—H4TA	0.9900
N1B—C5B	1.348 (6)	C4TH—H4TB	0.9900
			
N4A—Zn1A—N2A	145.65 (19)	C20B—N3B—Zn1B	110.4 (3)
N4A—Zn1A—N3A	84.49 (17)	C16B—N3B—Zn1B	131.5 (4)
N2A—Zn1A—N3A	117.97 (18)	C21B—N4B—Si2B	116.4 (4)
N4A—Zn1A—N1A	119.56 (18)	C21B—N4B—Zn1B	114.3 (3)
N2A—Zn1A—N1A	83.91 (17)	Si2B—N4B—Zn1B	129.3 (2)
N3A—Zn1A—N1A	99.84 (16)	N1B—C1B—C2B	123.1 (5)
N2A—Si1A—C13A	107.1 (3)	N1B—C1B—H1BA	118.4
N2A—Si1A—C7A	109.6 (2)	C2B—C1B—H1BA	118.4
C13A—Si1A—C7A	114.0 (3)	C1B—C2B—C3B	118.3 (5)
N2A—Si1A—C10A	112.8 (3)	C1B—C2B—H2BA	120.8
C13A—Si1A—C10A	107.6 (3)	C3B—C2B—H2BA	120.8
C7A—Si1A—C10A	105.9 (3)	C4B—C3B—C2B	118.4 (5)
N4A—Si2A—C25A	115.0 (2)	C4B—C3B—H3BA	120.8
N4A—Si2A—C28A	105.5 (2)	C2B—C3B—H3BA	120.8
C25A—Si2A—C28A	107.6 (3)	C3B—C4B—C5B	120.3 (5)
N4A—Si2A—C22A	110.8 (3)	C3B—C4B—H4BA	119.9
C25A—Si2A—C22A	104.7 (3)	C5B—C4B—H4BA	119.9
C28A—Si2A—C22A	113.5 (3)	N1B—C5B—C4B	120.8 (5)
C5A—N1A—C1A	118.9 (5)	N1B—C5B—C6B	117.4 (4)
C5A—N1A—Zn1A	109.9 (4)	C4B—C5B—C6B	121.8 (5)
C1A—N1A—Zn1A	131.2 (4)	N2B—C6B—C5B	113.3 (4)
C6A—N2A—Si1A	118.1 (4)	N2B—C6B—H6BA	108.9
C6A—N2A—Zn1A	114.2 (3)	C5B—C6B—H6BA	108.9
Si1A—N2A—Zn1A	127.7 (2)	N2B—C6B—H6BB	108.9
C16A—N3A—C20A	120.2 (5)	C5B—C6B—H6BB	108.9
C16A—N3A—Zn1A	130.4 (4)	H6BA—C6B—H6BB	107.7
C20A—N3A—Zn1A	109.4 (3)	C8B—C7B—C9B	109.6 (5)
C21A—N4A—Si2A	118.3 (3)	C8B—C7B—Si1B	114.2 (4)
C21A—N4A—Zn1A	114.4 (3)	C9B—C7B—Si1B	113.6 (4)
Si2A—N4A—Zn1A	127.3 (2)	C8B—C7B—H7BA	106.2
N1A—C1A—C2A	122.4 (6)	C9B—C7B—H7BA	106.2
N1A—C1A—H1AA	118.8	Si1B—C7B—H7BA	106.2
C2A—C1A—H1AA	118.8	C7B—C8B—H8BA	109.5
C1A—C2A—C3A	119.5 (5)	C7B—C8B—H8BB	109.5
C1A—C2A—H2AA	120.3	H8BA—C8B—H8BB	109.5
C3A—C2A—H2AA	120.3	C7B—C8B—H8BC	109.5
C2A—C3A—C4A	118.9 (5)	H8BA—C8B—H8BC	109.5
C2A—C3A—H3AA	120.5	H8BB—C8B—H8BC	109.5
C4A—C3A—H3AA	120.5	C7B—C9B—H9BA	109.5
C5A—C4A—C3A	118.8 (5)	C7B—C9B—H9BB	109.5
C5A—C4A—H4AA	120.6	H9BA—C9B—H9BB	109.5
C3A—C4A—H4AA	120.6	C7B—C9B—H9BC	109.5
N1A—C5A—C4A	121.5 (5)	H9BA—C9B—H9BC	109.5
N1A—C5A—C6A	117.0 (5)	H9BB—C9B—H9BC	109.5
C4A—C5A—C6A	121.5 (5)	C12B—C10B—C11B	108.4 (6)
N2A—C6A—C5A	114.8 (5)	C12B—C10B—Si1B	112.4 (4)
N2A—C6A—H6AA	108.6	C11B—C10B—Si1B	114.0 (4)
C5A—C6A—H6AA	108.6	C12B—C10B—H10B	107.3
N2A—C6A—H6AB	108.6	C11B—C10B—H10B	107.3
C5A—C6A—H6AB	108.6	Si1B—C10B—H10B	107.3
H6AA—C6A—H6AB	107.5	C10B—C11B—H11D	109.5
C9A—C7A—C8A	111.3 (6)	C10B—C11B—H11E	109.5
C9A—C7A—Si1A	113.6 (4)	H11D—C11B—H11E	109.5
C8A—C7A—Si1A	114.2 (4)	C10B—C11B—H11F	109.5
C9A—C7A—H7AA	105.6	H11D—C11B—H11F	109.5
C8A—C7A—H7AA	105.6	H11E—C11B—H11F	109.5
Si1A—C7A—H7AA	105.6	C10B—C12B—H12D	109.5
C7A—C8A—H8AA	109.5	C10B—C12B—H12E	109.5
C7A—C8A—H8AB	109.5	H12D—C12B—H12E	109.5
H8AA—C8A—H8AB	109.5	C10B—C12B—H12F	109.5
C7A—C8A—H8AC	109.5	H12D—C12B—H12F	109.5
H8AA—C8A—H8AC	109.5	H12E—C12B—H12F	109.5
H8AB—C8A—H8AC	109.5	C15B—C13B—C14B	109.9 (6)
C7A—C9A—H9AA	109.5	C15B—C13B—Si1B	113.6 (4)
C7A—C9A—H9AB	109.5	C14B—C13B—Si1B	115.6 (4)
H9AA—C9A—H9AB	109.5	C15B—C13B—H13B	105.6
C7A—C9A—H9AC	109.5	C14B—C13B—H13B	105.6
H9AA—C9A—H9AC	109.5	Si1B—C13B—H13B	105.6
H9AB—C9A—H9AC	109.5	C13B—C14B—H14D	109.5
C11A—C10A—C12A	109.7 (7)	C13B—C14B—H14E	109.5
C11A—C10A—Si1A	111.6 (5)	H14D—C14B—H14E	109.5
C12A—C10A—Si1A	114.9 (5)	C13B—C14B—H14F	109.5
C11A—C10A—H10A	106.7	H14D—C14B—H14F	109.5
C12A—C10A—H10A	106.7	H14E—C14B—H14F	109.5
Si1A—C10A—H10A	106.7	C13B—C15B—H15D	109.5
C10A—C11A—H11A	109.5	C13B—C15B—H15E	109.5
C10A—C11A—H11B	109.5	H15D—C15B—H15E	109.5
H11A—C11A—H11B	109.5	C13B—C15B—H15F	109.5
C10A—C11A—H11C	109.5	H15D—C15B—H15F	109.5
H11A—C11A—H11C	109.5	H15E—C15B—H15F	109.5
H11B—C11A—H11C	109.5	N3B—C16B—C17B	123.9 (5)
C10A—C12A—H12A	109.5	N3B—C16B—H16B	118.1
C10A—C12A—H12B	109.5	C17B—C16B—H16B	118.1
H12A—C12A—H12B	109.5	C18B—C17B—C16B	117.6 (6)
C10A—C12A—H12C	109.5	C18B—C17B—H17B	121.2
H12A—C12A—H12C	109.5	C16B—C17B—H17B	121.2
H12B—C12A—H12C	109.5	C19B—C18B—C17B	119.9 (6)
C15A—C13A—C14A	107.5 (6)	C19B—C18B—H18B	120.1
C15A—C13A—Si1A	115.9 (5)	C17B—C18B—H18B	120.1
C14A—C13A—Si1A	114.8 (5)	C18B—C19B—C20B	120.0 (5)
C15A—C13A—H13A	105.9	C18B—C19B—H19B	120.0
C14A—C13A—H13A	105.9	C20B—C19B—H19B	120.0
Si1A—C13A—H13A	105.9	N3B—C20B—C19B	120.5 (5)
C13A—C14A—H14A	109.5	N3B—C20B—C21B	117.4 (5)
C13A—C14A—H14B	109.5	C19B—C20B—C21B	122.1 (5)
H14A—C14A—H14B	109.5	N4B—C21B—C20B	113.7 (5)
C13A—C14A—H14C	109.5	N4B—C21B—H21C	108.8
H14A—C14A—H14C	109.5	C20B—C21B—H21C	108.8
H14B—C14A—H14C	109.5	N4B—C21B—H21D	108.8
C13A—C15A—H15A	109.5	C20B—C21B—H21D	108.8
C13A—C15A—H15B	109.5	H21C—C21B—H21D	107.7
H15A—C15A—H15B	109.5	C23B—C22B—C24B	107.5 (5)
C13A—C15A—H15C	109.5	C23B—C22B—Si2B	115.1 (4)
H15A—C15A—H15C	109.5	C24B—C22B—Si2B	112.6 (4)
H15B—C15A—H15C	109.5	C23B—C22B—H22B	107.1
N3A—C16A—C17A	122.9 (6)	C24B—C22B—H22B	107.1
N3A—C16A—H16A	118.6	Si2B—C22B—H22B	107.1
C17A—C16A—H16A	118.6	C22B—C23B—H23D	109.5
C18A—C17A—C16A	117.3 (6)	C22B—C23B—H23E	109.5
C18A—C17A—H17A	121.4	H23D—C23B—H23E	109.5
C16A—C17A—H17A	121.4	C22B—C23B—H23F	109.5
C19A—C18A—C17A	120.1 (6)	H23D—C23B—H23F	109.5
C19A—C18A—H18A	119.9	H23E—C23B—H23F	109.5
C17A—C18A—H18A	119.9	C22B—C24B—H24D	109.5
C18A—C19A—C20A	119.2 (5)	C22B—C24B—H24E	109.5
C18A—C19A—H19A	120.4	H24D—C24B—H24E	109.5
C20A—C19A—H19A	120.4	C22B—C24B—H24F	109.5
N3A—C20A—C19A	120.3 (5)	H24D—C24B—H24F	109.5
N3A—C20A—C21A	118.4 (5)	H24E—C24B—H24F	109.5
C19A—C20A—C21A	121.2 (5)	C26B—C25B—C27B	111.8 (5)
N4A—C21A—C20A	113.0 (4)	C26B—C25B—Si2B	111.8 (4)
N4A—C21A—H21A	109.0	C27B—C25B—Si2B	118.1 (4)
C20A—C21A—H21A	109.0	C26B—C25B—H25B	104.6
N4A—C21A—H21B	109.0	C27B—C25B—H25B	104.6
C20A—C21A—H21B	109.0	Si2B—C25B—H25B	104.6
H21A—C21A—H21B	107.8	C25B—C26B—H26D	109.5
C23A—C22A—C24A	111.9 (5)	C25B—C26B—H26E	109.5
C23A—C22A—Si2A	114.1 (5)	H26D—C26B—H26E	109.5
C24A—C22A—Si2A	113.6 (4)	C25B—C26B—H26F	109.5
C23A—C22A—H22A	105.4	H26D—C26B—H26F	109.5
C24A—C22A—H22A	105.4	H26E—C26B—H26F	109.5
Si2A—C22A—H22A	105.4	C25B—C27B—H27D	109.5
C22A—C23A—H23A	109.5	C25B—C27B—H27E	109.5
C22A—C23A—H23B	109.5	H27D—C27B—H27E	109.5
H23A—C23A—H23B	109.5	C25B—C27B—H27F	109.5
C22A—C23A—H23C	109.5	H27D—C27B—H27F	109.5
H23A—C23A—H23C	109.5	H27E—C27B—H27F	109.5
H23B—C23A—H23C	109.5	C29B—C28B—C30B	109.6 (5)
C22A—C24A—H24A	109.5	C29B—C28B—Si2B	115.8 (4)
C22A—C24A—H24B	109.5	C30B—C28B—Si2B	114.5 (4)
H24A—C24A—H24B	109.5	C29B—C28B—H28B	105.3
C22A—C24A—H24C	109.5	C30B—C28B—H28B	105.3
H24A—C24A—H24C	109.5	Si2B—C28B—H28B	105.3
H24B—C24A—H24C	109.5	C28B—C29B—H29D	109.5
C26A—C25A—C27A	106.7 (5)	C28B—C29B—H29E	109.5
C26A—C25A—Si2A	114.5 (4)	H29D—C29B—H29E	109.5
C27A—C25A—Si2A	111.3 (4)	C28B—C29B—H29F	109.5
C26A—C25A—H25A	108.0	H29D—C29B—H29F	109.5
C27A—C25A—H25A	108.0	H29E—C29B—H29F	109.5
Si2A—C25A—H25A	108.0	C28B—C30B—H30D	109.5
C25A—C26A—H26A	109.5	C28B—C30B—H30E	109.5
C25A—C26A—H26B	109.5	H30D—C30B—H30E	109.5
H26A—C26A—H26B	109.5	C28B—C30B—H30F	109.5
C25A—C26A—H26C	109.5	H30D—C30B—H30F	109.5
H26A—C26A—H26C	109.5	H30E—C30B—H30F	109.5
H26B—C26A—H26C	109.5	C6T—C1T—C2T	117.2 (10)
C25A—C27A—H27A	109.5	C6T—C1T—C7T	119.8 (13)
C25A—C27A—H27B	109.5	C2T—C1T—C7T	123.0 (12)
H27A—C27A—H27B	109.5	C3T—C2T—C1T	120.9 (11)
C25A—C27A—H27C	109.5	C3T—C2T—H2TC	119.5
H27A—C27A—H27C	109.5	C1T—C2T—H2TC	119.5
H27B—C27A—H27C	109.5	C2T—C3T—C4T	122.3 (11)
C30A—C28A—C29A	109.4 (5)	C2T—C3T—H3TC	118.8
C30A—C28A—Si2A	112.8 (4)	C4T—C3T—H3TC	118.8
C29A—C28A—Si2A	116.1 (4)	C3T—C4T—C5T	118.5 (11)
C30A—C28A—H28A	105.9	C3T—C4T—H4TC	120.7
C29A—C28A—H28A	105.9	C5T—C4T—H4TC	120.7
Si2A—C28A—H28A	105.9	C4T—C5T—C6T	119.3 (10)
C28A—C29A—H29A	109.5	C4T—C5T—H5TA	120.3
C28A—C29A—H29B	109.5	C6T—C5T—H5TA	120.3
H29A—C29A—H29B	109.5	C1T—C6T—C5T	121.5 (11)
C28A—C29A—H29C	109.5	C1T—C6T—H6TA	119.3
H29A—C29A—H29C	109.5	C5T—C6T—H6TA	119.3
H29B—C29A—H29C	109.5	C1T—C7T—H7TA	109.5
C28A—C30A—H30A	109.5	C1T—C7T—H7TB	109.5
C28A—C30A—H30B	109.5	H7TA—C7T—H7TB	109.5
H30A—C30A—H30B	109.5	C1T—C7T—H7TC	109.5
C28A—C30A—H30C	109.5	H7TA—C7T—H7TC	109.5
H30A—C30A—H30C	109.5	H7TB—C7T—H7TC	109.5
H30B—C30A—H30C	109.5	C4TH—O1TH—C1TH	107.6 (11)
N4B—Zn1B—N2B	143.02 (19)	C2TH—C1TH—O1TH	105.2 (12)
N4B—Zn1B—N1B	120.98 (18)	C2TH—C1TH—H1TA	110.7
N2B—Zn1B—N1B	83.74 (16)	O1TH—C1TH—H1TA	110.7
N4B—Zn1B—N3B	83.69 (17)	C2TH—C1TH—H1TB	110.7
N2B—Zn1B—N3B	122.00 (18)	O1TH—C1TH—H1TB	110.7
N1B—Zn1B—N3B	98.73 (17)	H1TA—C1TH—H1TB	108.8
N2B—Si1B—C10B	114.9 (2)	C1TH—C2TH—C3TH	109.7 (11)
N2B—Si1B—C13B	105.8 (2)	C1TH—C2TH—H2TA	109.7
C10B—Si1B—C13B	107.3 (3)	C3TH—C2TH—H2TA	109.7
N2B—Si1B—C7B	110.1 (2)	C1TH—C2TH—H2TB	109.7
C10B—Si1B—C7B	105.1 (3)	C3TH—C2TH—H2TB	109.7
C13B—Si1B—C7B	113.8 (3)	H2TA—C2TH—H2TB	108.2
N4B—Si2B—C28B	106.5 (2)	C4TH—C3TH—C2TH	113.1 (11)
N4B—Si2B—C22B	110.9 (2)	C4TH—C3TH—H3TA	109.0
C28B—Si2B—C22B	113.4 (3)	C2TH—C3TH—H3TA	109.0
N4B—Si2B—C25B	108.9 (2)	C4TH—C3TH—H3TB	109.0
C28B—Si2B—C25B	110.4 (3)	C2TH—C3TH—H3TB	109.0
C22B—Si2B—C25B	106.7 (3)	H3TA—C3TH—H3TB	107.8
C1B—N1B—C5B	119.1 (5)	C3TH—C4TH—O1TH	104.5 (11)
C1B—N1B—Zn1B	130.8 (4)	C3TH—C4TH—H4TA	110.9
C5B—N1B—Zn1B	110.1 (3)	O1TH—C4TH—H4TA	110.9
C6B—N2B—Si1B	119.2 (3)	C3TH—C4TH—H4TB	110.9
C6B—N2B—Zn1B	113.9 (3)	O1TH—C4TH—H4TB	110.9
Si1B—N2B—Zn1B	126.9 (2)	H4TA—C4TH—H4TB	108.9
C20B—N3B—C16B	118.1 (5)		
